# Multifocal muscle candidiasis of the legs in a patient with acute myeloid leukemia: A case report

**DOI:** 10.1097/MD.0000000000014580

**Published:** 2019-02-22

**Authors:** Ping Yi, Xiang Yang, MeiFang Yang, Yan Zhang, Lanjuan Li

**Affiliations:** a State Key Laboratory for Diagnosis and Treatment of Infectious Diseases, Collaborative Innovation Center for Diagnosis and Treatment of Infectious Diseases, The First Affiliated Hospital, College of Medicine, Zhejiang University; b Shu Lan (Hangzhou) Hospital, Hangzhou, China.

**Keywords:** acute myeloid leukemia, candida tropicalis, invasive candidal infections, muscle abscesses

## Abstract

**Rationale:**

Opportunistic infections frequently develop in immunocompromised patients, such as those with hematological malignancies, causing significant mortality. Early diagnosis of invasive fungal infections is often important and difficult due to the difficult nature of confirming infection using cytologic and histologic findings. However, we report the first case of candidal infection leading to muscle abscesses in the legs of a patient with leukemia.

**Patient concerns:**

A 60-year-old man with acute myeloid leukemia (AML) presented with multifocal muscle abscesses of the legs.

**Diagnoses:**

Multifocal muscle candidiasis of the legs was confirmed by fine-needle aspiration biopsy of 2 of the calf lesions.

**Interventions:**

After treatment with amphotericin B and flucytosine for 1 month, the patient was administered intravenous caspofungin for 3 months.

**Outcome:**

A CT scan of the abdomen and an MRI of the lower calves showed significant improvement.

**Lessons:**

This case highlights that fungal infection should be considered when patients present with multiple abscesses, emphasizing the value of early biopsy to confirm diagnosis and facilitate precision treatment.

## Abstract 2

Supplemental Digital Content is available in the text

## 1 Introduction

Opportunistic infections frequently develop in immunocompromised patients, such as those with hematological malignancies, causing significant mortality.^[1]^ Invasive candidal infections (ICIs) are a common cause of death in these patients.^[2]^ Early diagnosis of ICIs is important and often difficult due to the difficult nature of confirming infection using cytologic and histologic findings.^[3]^ Treatment is dependent on the patient receiving the full dosage and course of antifungal agents according to the antimicrobial susceptibility test. Herein, we describe a case of a patient with acute myeloid leukemia (AML M5 CBFB-MYH11+) who presented with multifocal muscle candidiasis of the legs (MMC) and pulmonary candidiasis (PC) during postremission consolidation chemotherapy. Although increasing antifungal drugs are clinically available, increasing rates of resistance and unknown courses of treatment limit the survival of these patients.

## 2 Case report

**Figure 1:**
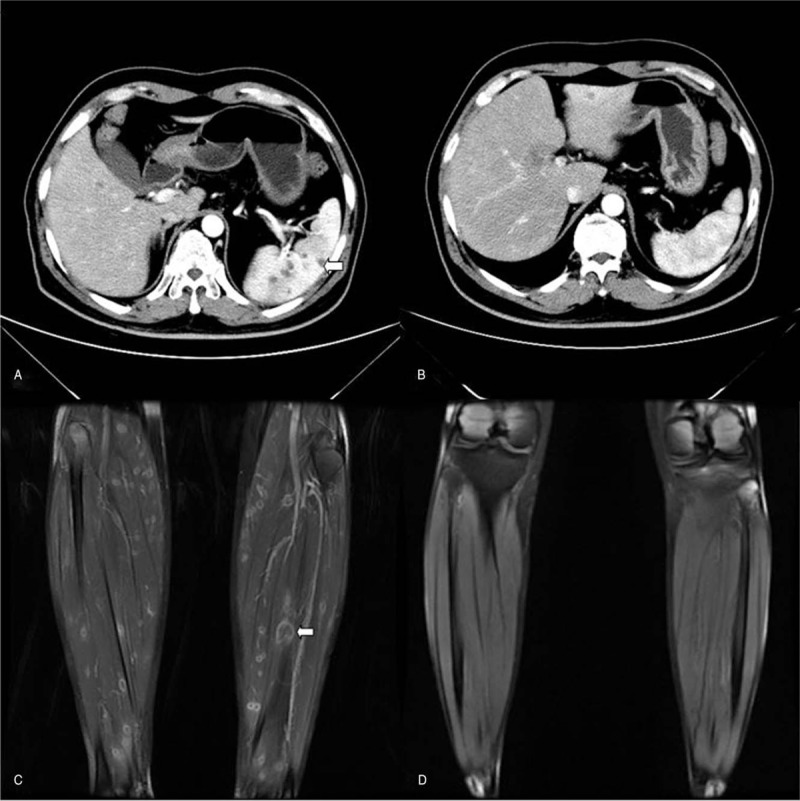
Imaging findings of spleen and legs before and after antifungal therapy (A) CT scan at initial showed a few hypodensities in the spleen; (B) Latest CT scan done at 2 months post antifungal treatment presenting improvement in the splenic lesions; (C) Magnetic resonance imaging scan after neutrophil recovery post chemotherapy showed worsening radiologic hypodensities within the calves; (D) Magnetic resonance imaging scan after antifungal treatment showed improving radiologic hypodensities within the calves. CT = computed tomography.

A 60-year-old Chinese man with a case of acute granulo-monocytic leukemia in remission after the fifth myelosuppressive chemotherapy was admitted to the infection ward with a 20-day history of fever and swelling of the calves. The relevant history revealed that the patient was diagnosed with AML in July 2017. He was subsequently treated with chemotherapy from July 2017 to February 2018 and acquired complete remission-induction. After the latest period of chemotherapy in February 2018 (day 0), the patient presented with neutropenic fever and swelling of the lower limbs (day 15), and his fever continued despite broad-spectrum antimicrobial and voriconazole therapy, which was used as antifungal prophylaxis. A routine computed tomography (CT) scan of the abdomen and thorax on day 26 of chemotherapy was examined and revealed multiple hypodense lesions in the spleen (Fig. 1A) and right lung. The patient was continually treated with broad-spectrum antimicrobial and voriconazole therapy. After neutrophil recovery 3 days later (day 31), the patient requested discharge from the hospital and had low-grade fever and swelling of the limbs.

Unfortunately, the patient was subsequently readmitted to the hospital with high fever and severe swelling of the limbs on day 39. Multiple small nodules were assessed throughout both calves, and we found multiple subcutaneous abscesses in the calves as shown by B-ultrasound examination and magnetic resonance imaging (MRI) (Fig. 1C), which were suggestive of a possible infection. Meanwhile, fine-needle aspiration biopsy of 2 of the calf lesions revealed the presence of *Candida tropicalis* (sensitive to amphotericin and flucytosine, resistant to fluconazole, itraconazole, and voriconazole) (Supplementary Fig. 1). A lumbar puncture was performed to assess for central nervous system fungal infection, which revealed that the cerebrospinal fluid was negative for Candida. No nucleated cells were found in the cerebrospinal fluid. In addition, protein levels, serum glucose, and glucose levels were normal.

Due to the urgency needed in treating ICIs, antifungal therapy with amphotericin B for 6 to 8 hours daily (1 mg/kg/day) and flucytosine (at a dose of 2.5 g every 8 hours) was initiated according to the drug sensitivity test. Fine-needle aspiration of one of the hepatic lesions was scheduled 1 month after treatment with amphotericin B and flucytosine but was not performed because the patient had improved after treatment and was without fever, exhibiting normal alkaline phosphatase and improved C-reactive protein levels along with reduced swelling of his limbs. A repeat CT scan of the abdomen and lower limbs after 1 month of amphotericin B and flucytosine therapy revealed stable hepatosplenic and muscle lesions. Considering the side effects of amphotericin B, caspofungin was continued for 1 month at which time CT scan of the abdomen (Fig. 1B) and MRI (Fig. 1D) of the lower calves showed improvement.

## 3 Discussion

Candida is the most common cause of fungal infections in humans. Many species are harmless to their hosts, including humans. However, when mucosal barriers are disrupted or the immune system is compromised, they can invade and cause disease. Candida species are the fourth most common pathogens isolated in blood cultures in the United States.^[4]^

ICIs cause increased morbidity and significant mortality among patients with hematological malignancies attributed to broad-spectrum antibiotics, postchemotherapy neutropenia, and venous catheterization.^[5,6]^ Isolation of the fungus through the culture of biological fluids and tissues is the current gold standard to diagnose invasive fungal infections.^[7]^ However, it is difficult to confirm a positive infection using cytologic and histologic findings. The prophase of antifungal therapy prior to biopsy may result in negative pathological findings. In this case, we achieved positive *C tropicalis* cultures from the biopsy of 2 small nodules in the swollen leg. Meanwhile, we performed drug sensitivity analysis, demonstrating that *C tropicalis* was resistant to triazole antifungal agents, such as fluconazole, itraconazole, and voriconazole, but was sensitive to amphotericin and flucytosine. These drug sensitivity results could lead to the early implementation of accurate antifungal therapy, improving the recovery rate.

The triazoles fluconazole, itraconazole, voriconazole, and posaconazole are widely recognized drugs for Candida species infections due to their efficacy and low toxicity.^[7]^ Given their safety profiles, they are used for fungal infections and as prophylactic treatments in high-risk patients. However, the widespread use of antifungal agents over the last 2 decades has contributed to the development of antifungal resistance.^[8]^ In our case, prophylactic voriconazole treatment resulted in an early exposure to antifungal agents, which might be a major driving factor for the development of resistance to triazoles. The presence of resistance to triazole antifungal agents, including fluconazole, itraconazole, and voriconazole, in our patient caused a substantial therapeutic challenge, as antifungal drugs were limited and as the pathogen only exhibited sensitivity to amphotericin B and caspofungin in our patient. Unfortunately, the former has significant toxicity and side effects, and the latter is cost prohibitive. Due to the long-term treatment of invasive *C tropicalis*, it is necessary to administer oral antifungal agents for a long time after the disease is stable. However, the patient was resistant to triazole drugs, which can be administered orally at present. Our patient needed a long-term injection treatment, which would increase both the economic burden to the patient and the possibility of nosocomial infection.

The muscle lesions in this case presented as well-circumscribed, multifocal, nodule lesions. We consider this as the first reported case of candidal infection leading to multifocal muscle lesions in an immunodepressed patient with hematologic malignancy. The atypical manifestations of multifocal myalgia and subcutaneous masses, in this case, should arouse the vigilance of clinicians to other possible clinical presentations of candidal infection besides the common PC, HSC, and septic arthritis described in the literature of hematologic malignancy patients.^[9,10]^ However, this case differs from others in that despite a diagnosis and therapy, the course of treatment was not defined.

In summary, we report a rare case of multiple subcutaneous abscesses of the lower limb caused by *C tropicalis*. We draw attention to the fact that fungal infections must be fully considered in patients with immune deficiency presenting with multiple subcutaneous abscesses of the lower limb without venous catheterization. We emphasize the value of early biopsy of the abscesses to confirm the diagnosis and facilitate early treatment. Additionally, we highlight that the most significant factors for the successful treatment of fungal ICIs are early diagnosis and prompt individualized therapy based on drug sensitivity results, appropriate dosing, and course of treatment.

## Acknowledgments

We thank all patients and clinicians for their participation. We thank Wang Junli (Radiologists, the First Affiliated Hospital, College of Medicine, Zhejiang University) for providing MRI and CT data.

## Author contributions

**Data curation:** Ping Yi, MeiFang Yang.

**Formal analysis:** Ping Yi, Xiang Yang. Yan Zhang

**Funding acquisition:** Lanjuan Li.

**Investigation:** Ping Yi, Xiang Yang.

**Project administration:** Ping Yi, Xiang Yang, Lanjuan Li

**Supervision:** Lanjuan Li, MeiFang Yang.

**Writing – original draft:** Ping Yi.

**Writing – review & editing:** Lanjuan Li.

## Supplementary Material

Supplemental Digital Content
