# Supplementary material for: Multifocal muscle candidiasis of the legs in a patient with acute myeloid leukemia: A case report
Source: Medicine (Baltimore). 2019 Feb 22;98(8):e14580. doi: 10.1097/MD.0000000000014580 (PMC6408043; doi:10.1097/MD.0000000000014580)
Supplement: Supplemental Digital Content [file medi-98-e14580-s001.doc]

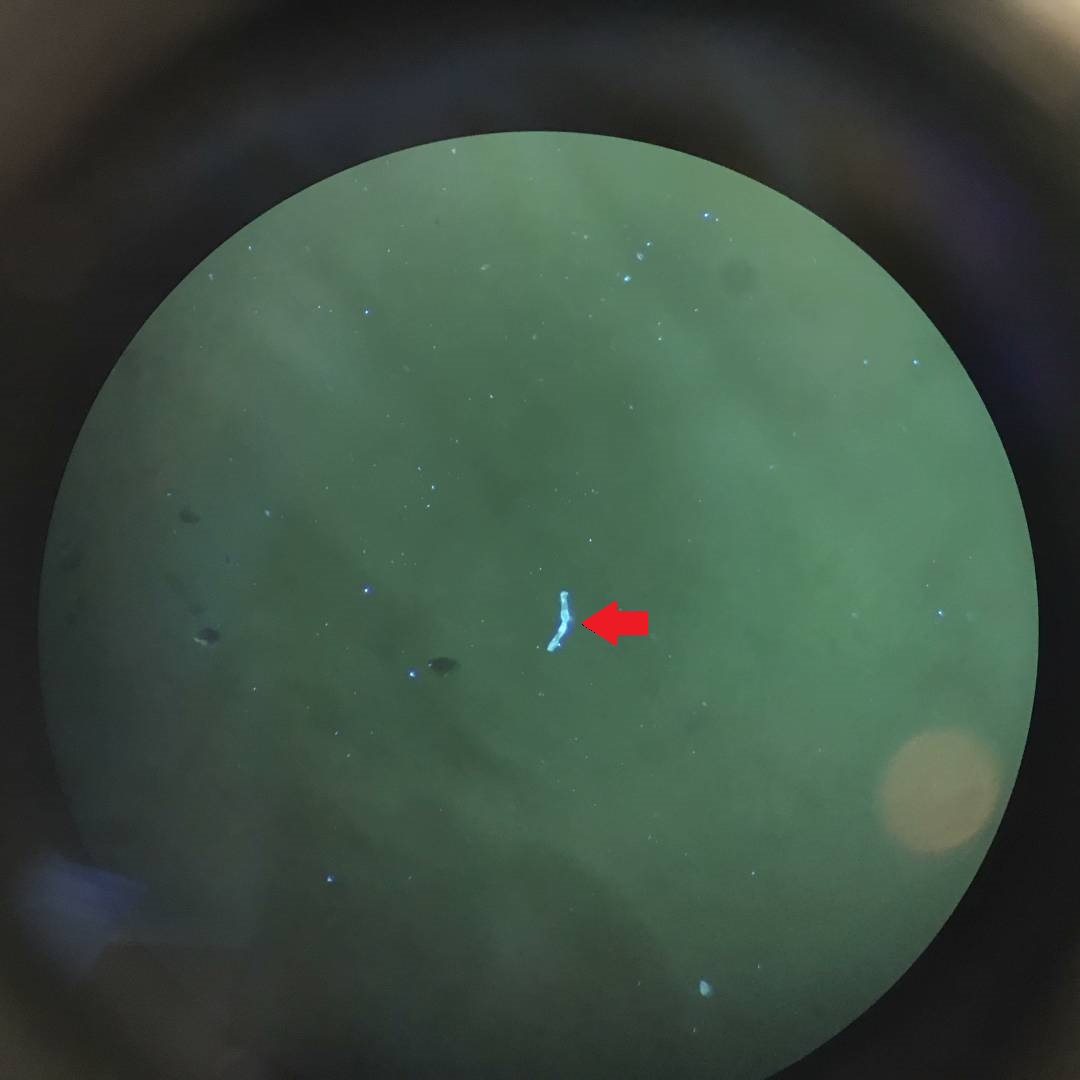


**Supplementary Figure 1** Fine-needle aspiration biopsy of two of the calf lesions presented that Candida tropicalis was subsequently isolated: the arrow presents a [symbolic](http://www.iciba.com/symbolic) fungal structure
